# Gallic acid attenuates LPS-induced inflammation in Caco-2 cells by suppressing the activation of the NF-κB/MAPK signaling pathway: Gallic acid alleviates the inflammation in Caco-2 cells

**DOI:** 10.3724/abbs.2024008

**Published:** 2024-03-22

**Authors:** Chu Chu, Huan Ru, Yuyan Chen, Jinhua Xu, Caihong Wang, Yuanxiang Jin

**Affiliations:** College of Biotechnology and Bioengineering Zhejiang University of Technology Hangzhou 310032 China

**Keywords:** gallic acid, inflammatory bowel disease, tight junction protein, cell apoptosis, oxidative stress, NF-κB/MAPK pathway

## Abstract

Inflammatory bowel disease (IBD) is a chronic inflammatory disease characterized by intestinal barrier dysfunction, inflammatory synergistic effects and excessive tissue injury. Gallic acid (GA) is renowned for its remarkable biological activity, encompassing anti-inflammatory and antioxidant properties. However, the underlying mechanisms by which GA protects against intestinal inflammation have not been fully elucidated. The aim of this study is to investigate the effect of GA on the inflammation of a lipopolysaccharide (LPS)-stimulated human colon carcinoma cell line (Caco-2) and on the intestinal barrier dysfunction, and explore the underlying molecular mechanism involved. Our findings demonstrate that 5 μg/mL GA restores the downregulation of the mRNA and protein levels of Claudin-1, Occludin, and ZO-1 and decreases the expressions of inflammatory factors such as IL-6, IL-1β and TNF-α induced by LPS. In addition, GA exhibits a protective effect by reducing the LPS-enhanced early and late apoptotic ratios, downregulating the mRNA levels of pro-apoptotic factors ( *Bax*, *Bad*, *Caspase-3*, *Caspase-8*, and *Caspase-9*), and upregulating the mRNA levels of anti-apoptotic factor *Bcl-2* in Caco-2 cells. GA also reduces the levels of reactive oxygen species increased by LPS and restores the activity of antioxidant enzymes, namely, superoxide dismutase and catalase, as well as the level of glutathione. More importantly, GA exerts its anti-inflammatory effects by inhibiting the LPS-induced phosphorylation of key signaling molecules in the NF-κB/MAPK pathway, including p65, IκB-α, p38, JNK, and ERK, in Caco-2 cells. Overall, our findings show that GA increases the expressions of tight junction proteins, reduces cell apoptosis, relieves oxidative stress and suppresses the activation of the NF-κB/MAPK pathway to reduce LPS-induced intestinal inflammation in Caco-2 cells, indicating that GA has potential as a therapeutic agent for intestinal inflammation.

## Introduction

Inflammatory bowel disease (IBD), encompassing Crohn’s disease (CD) and ulcerative colitis (UC), manifests chronic inflammation and dysplasia of epithelial barrier function [ 1, 2]. An increasing incidence of IBD has been reported worldwide. Due to shifts in diet and lifestyle, the prevalence of IBD has been steadily increasing, especially in developing countries, in recent years [ 3, 4]. The pathogenesis of IBD may be related to the interaction of genetic, immune, infectious and psychiatric factors [ 5, 6]. The damage mediated by inflammation disrupts tight junction (TJ) proteins and increases paracellular permeability to microbes and antigens [ 7, 8]. Long-term damage to TJ proteins may lead to IBD [ 9, 10]. Consequently, remission of IBD necessitates a decrease in inflammatory responses and reinforcement of intestinal barrier integrity [11].

*In vivo* animal models are too complex to control many gut processes alone. Thus, new treatments for IBD have been developed in a variety of cell models to simulate intestinal inflammation. Caco-2 cells have emerged as valuable tools for studying intestinal diseases [ 12, 13]. Various drugs, such as 5-aminosalicylic acid drugs, steroids and immunosuppressants [14], have been used to treat IBD. However, the clinical treatment efficacy is unsatisfactory, and the side effects of long-term use are serious [14]. Therefore, there is an urgent need for some mild and effective treatment alternatives. Many studies have reported that plant phenols, including sinapic acid, have attracted increasing attention due to their antioxidant and anti-inflammatory properties and minimal harm to human health [ 15‒ 19]. These compounds act as antioxidants that inhibit the oxidation of DNA, proteins, lipids and enzymes linked to the production of free radicals [ 20‒ 22].

Gallic acid (GA), a plant polyphenol, is a naturally produced secondary metabolite that is present in a variety of fruits, plants, vegetables and nuts, such as strawberries, green tea and oak bark [ 23, 24]. GA has a low molecular weight and triphenolic structure, endowing it with potent anti-inflammatory and antioxidant capabilities [25]. The phenol hydroxyl group of GA can eliminate reactive oxygen species (ROS) and interrupt the cycle of new free radical formation. GA has anti-inflammatory effects by reducing proinflammatory mediators, inhibiting the expressions of nuclear transcription factors and downregulating downstream inflammatory targets [ 26, 27]. In addition to its anti-inflammatory effects, GA has been shown to have pharmacological effects on tumors, diabetes, and obesity [ 27‒ 29].

Despite its proven efficacy in treating various inflammation-related diseases, the effects and mechanism of action of GA in IBD remain unexplored. Therefore, in this study we used Caco-2 cells as an *in vitro* model to explore the effects of GA on the inflammatory response induced by lipopolysaccharide (LPS) and to investigate the possible underlying mechanisms involved.

## Materials and Methods

### Reagents

GA and LPS were obtained from Sigma-Aldrich (St Louis, USA). Assay kits for the detection of superoxide dismutase (SOD), glutathione (GSH) and catalase (CAT) were obtained from Jiangcheng Institute of Bioengineering (Nanjing, China). Antibodies against Claudin-1, Occludin, IL-1β and Toll-like receptor 4 (TLR4) were obtained from Cell Signaling Technology (Beverly, USA). Antibodies against NF-κB, IκB-α, p38, JNK, ERK, p-p65, p-IκB-α, p-p38, p-JNK, and p-ERK were purchased from Abcam (Cambridge, USA).

### Cell culture

Caco-2 cells obtained from the National Collection of Authenticated Cell Cultures (Shanghai, China) were cultured in high-glucose Dulbecco’s modified Eagle’s medium (DMEM; Biological Industries, Kibbutz Beit Haemek, Israel) supplemented with 1% penicillin-streptomycin (Beyotime, Haimen, China) and 10% fetal bovine serum (HAKATA, Shanghai, China) in an atmosphere of 5% CO _2_ at 37°C. The medium was refreshed every 2 days. Then, the cells were subcultured in 60.8-cm ^2^ cell culture dishes (BioFil, Guangzhou, China) at 80% confluence by using 0.25% trypsin-EDTA solution (Biological Industries).

### Cell viability assay

The cytotoxicity of GA and LPS to Caco-2 cells was evaluated by cell counting kit-8 (CCK-8) assay. Caco-2 cells were cultivated in 96-well plates at a density of 1‒2×10 ^4^ cells/well. The cells were incubated with LPS at various concentrations (1, 5, 10, 50, and 100 μg/mL) for 4 and 24 h when 70%‒80% of the Caco-2 cells adhered to the well. The cells were treated with GA at different concentrations (1, 5, 10, 20, 40, and 50 μg/mL) for 24 and 48 h respectively. After that, 100 μL of serum-free DMEM supplemented with high glucose and 10% CCK8 solution (Beyotime) was added to each well and incubated for 1‒2 h. A microplate reader (Allsheng, Hangzhou, China) was used to measure the absorbance at 450 nm.

### Immunofluorescence staining

Caco-2 cells were inoculated on coverslips in a 24-well plate. After indicated treatment, the cell samples were fixed with 4% paraformaldehyde at 4°C overnight. Then fixed cells were rinsed with PBS and permeabilized with 1 mL of 0.1% Triton X-100/PBS for 15‒20 min. Then, the cells were washed 3 times with PBS and blocked with 5% fetal bovine serum (FBS) in PBS. After rinsing once again with PBS, the cells were incubated with primary antibodies overnight at 4°C, followed by incubation with Alexa Fluor® 488-labelled secondary antibodies for 1 h. Caco-2 cells were observed with a fluorescence microscope (Nikon, Tokyo, Japan). At least 5 visual fields were randomly selected for each slide for imaging.

### Quantitative reverse-transcription (qRT)-PCR

After treatment, the collected cell samples were lysed and total RNA was extracted using Trizol reagent (Invitrogen, Carlsbad, USA). The total RNA was reverse-transcribed into cDNA using reverse transcriptase (Vazyme, Nanjing, China). Subsequently, the cDNA was subject to qPCR. qRT-PCR was performed on a CFX Connect ^TM^ Real-Time System (Bio-Rad Laboratories, Hercules, USA) with the following procedure: 1 min at 95°C, followed by 40 cycles of 15 s at 95°C and 1 min at 60°C. The total volume of every reaction was 10 μL in a 96-well plate, which included 1 μL of cDNA, 5 μL of 2× ChamQ Universal SYBR qPCR Master Mix (Vazyme), and 0.4 μL of forward and reverse primers, and double distilled water. The primers used were synthesized by Sangon Biotech (Shanghai, China) and the sequence information is shown in Supplementary Table S1.

### Western blot analysis

RIPA lysis buffer was used for extraction of total protein from Caco-2 cells, and the protein concentration was determined using a BCA protein assay kit (Thermo Fisher Scientific, Shanghai, China). Then, 20‒30 μg of protein was separated via SDS-PAGE and subsequently transferred to polyvinylidene difluoride (PVDF) membranes (Millipore, Billerica, USA). The membranes were blocked with Protein Free Rapid Blocking Buffer (Epizyme, Shanghai, China) at room temperature for 15 min, and then incubated with specific primary antibodies at 4°C overnight. After washing 3 times using Tris-buffered saline supplemented with Tween® 20 (TBST), the membranes were incubated with the appropriate horseradish peroxidase (HRP)-conjugated secondary antibodies for 1 h at room temperature. After extensive wash, the protein bands were visualized via enhanced chemiluminescence (ECL) reagent (Haoke, Hangzhou, China) and quantified via ImageJ Pro-Plus 6.0 (Tanon, Shanghai, China).

### Measurement of intracellular ROS

An ROS detection kit (Beyotime) was used to determine the intracellular ROS level. Briefly, Caco-2 cells were seeded into 24-well plates. Once the cells reached 70%‒80% confluence, they were treated with varying concentrations of GA (1, 5, or 10 μg/mL) for 24 h, followed by stimulation with LPS (10 μg/mL) for 4 h. Then, diluted DCFH-DA (10 μM) was added to the wells and incubated at 37°C for 30 min. Finally, fluorescence images were captured with a fluorescence microscope and fluorescence intensity was quantified using ImageJ Pro-Plus 6.0 (Tanon). The fluorescence intensity reflects intracellular ROS level.

### Determination of antioxidant parameters

Caco-2 cells were seeded into 6-well plates. Once the cells reached 70%‒80% confluence, they were treated with varying concentrations of GA (1, 5, or 10 μg/mL) for 24 h, followed by stimulation with LPS (10 μg/mL) for 4 h. Then, RIPA lysis buffer was used for extraction of total protein from Caco-2 cells, and the protein concentration was determined using a BCA protein assay kit (Thermo Fisher Scientific). The activities of SOD, CAT and GSH were determined using kits (Jiancheng Bioengineering) via the addition of hydroxylamine, visible light and xanthopterin-oxidase-Verfahren, respectively. Finally, the samples to be tested were mixed with the assay reagents and added into the enzyme plate, incubated at 37°C for 20 min, and the OD values of SOD, CAT, and GSH were detected at 450 nm, 405 nm, and 412 nm, respectively, using a microplate reader.

### Cell apoptosis assay

Apoptosis in the drug-treated cells was detected by using a FITC Annexin V Apoptosis Detection Kit I (BD Pharmingen, Franklin Lakes, USA). Briefly, Caco-2 cells were inoculated into 6-well plates and cultured to more than 70%‒80% of confluence. Then, cells were pretreated with GA (10 μg/mL) for 24 h, and then stimulated with LPS (10 μg/mL) for 4 h. Caco-2 cells were trypsinized, washed twice with cold PBS, and then resuspended in fixative buffer. Subsequently, fixed cells were incubated with FITC-conjugated Annexin V (5 μL) and PI (5 μL) at 37 °C for 15 min in the dark. Finally, cell apoptosis was analyzed by flow cytometry.

### Statistical analysis

The data were presented as the mean±SEM. All the statistical analyses were performed with GraphPad Prism version 7.0 software. Two-way ANOVA and Tukey’s test were used to examine the differences among the control and other treatment groups. A *P* value less than 0.05 indicated statistical significance.

## Results

### Effects of LPS and GA on Caco-2 cell viability

**Figure 1:**
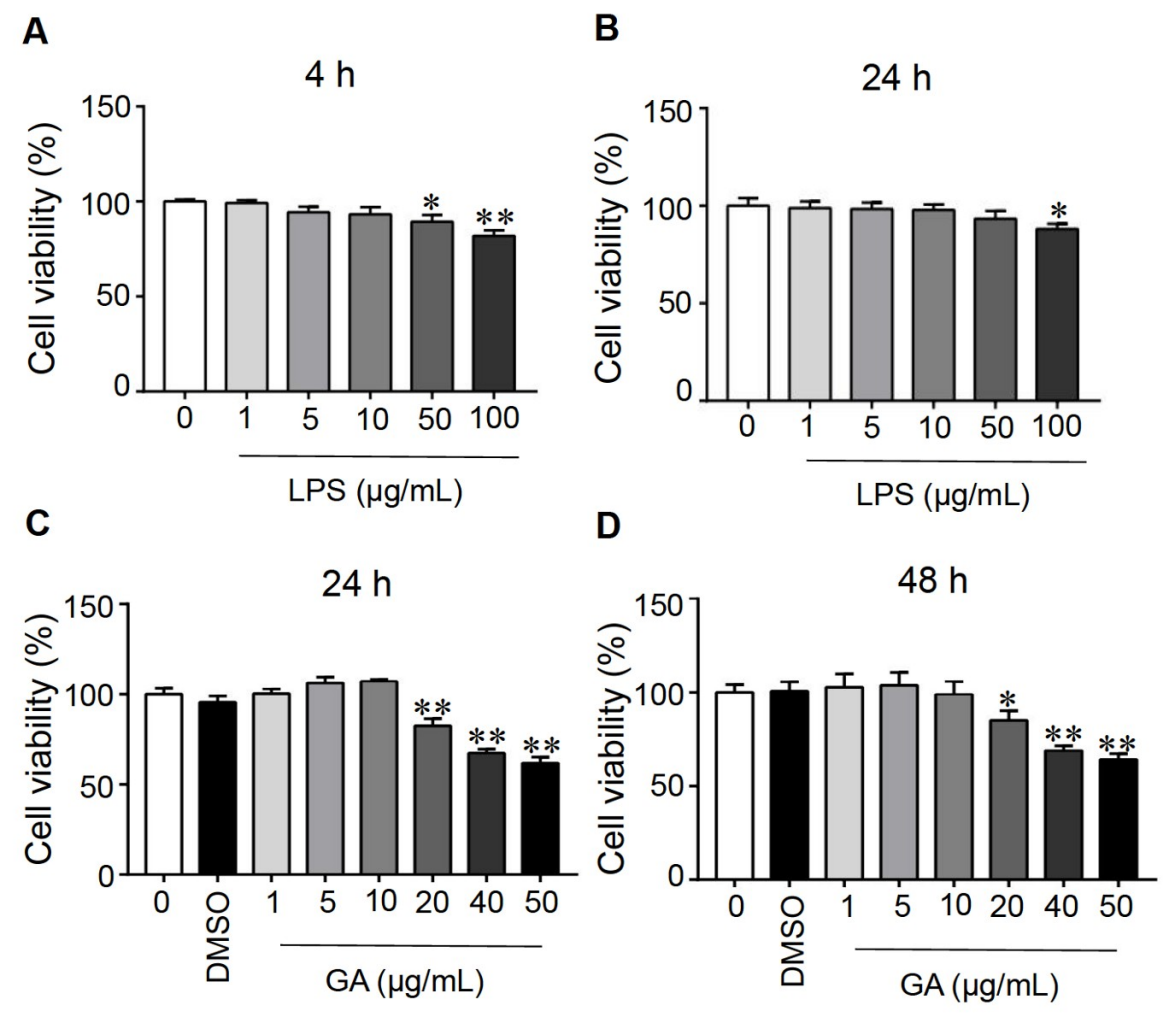
Figure 1 The influence of different concentrations of LPS and GA on Caco-2 cell viability After different concentrations of LPS were added to 96-well plates and coincubated with Caco-2 cells for (A) 4 h and (B) 24 h, the cell viability of the respective groups was inspected by a CCK-8 assay. Cell viability was assayed after Caco-2 cells were cultivated with GA at different concentrations for (C) 24 h and (D) 48 h. Data are presented as the mean±SEM of 6 separate experiments. Significant differences were found between means with different letters as determined by one-way ANOVA, n=6; *P<0.05 and **P<0.01 compared to the control. GA: gallic acid.

The viability of Caco-2 cells treated with LPS for 4 and 24 h at various concentrations (0, 1, 5, 10, 50, and 100 μg/mL) is shown in Figure 1A,B. The cell viability decreased when the LPS concentration was greater than 10 μg/mL, and it decreased more significantly at 4 h than at 24 h. Thus, in subsequent experiments, the cells were treated with 10 μg/mL LPS for 4 h. GA treatment at 20, 40 and 50 μg/mL for 24 and 48 h resulted in a substantial reduction in cell viability, while 1, 5, and 10 μg/mL GA exhibited no cytotoxicity to Caco-2 cells ( Figure 1C,D). Therefore, we selected GA at doses of 1, 5, and 10 μg/mL for 24 h for subsequent experiments.

### GA protected against LPS-induced cellular inflammatory response in Caco-2 cells

**Figure 2:**
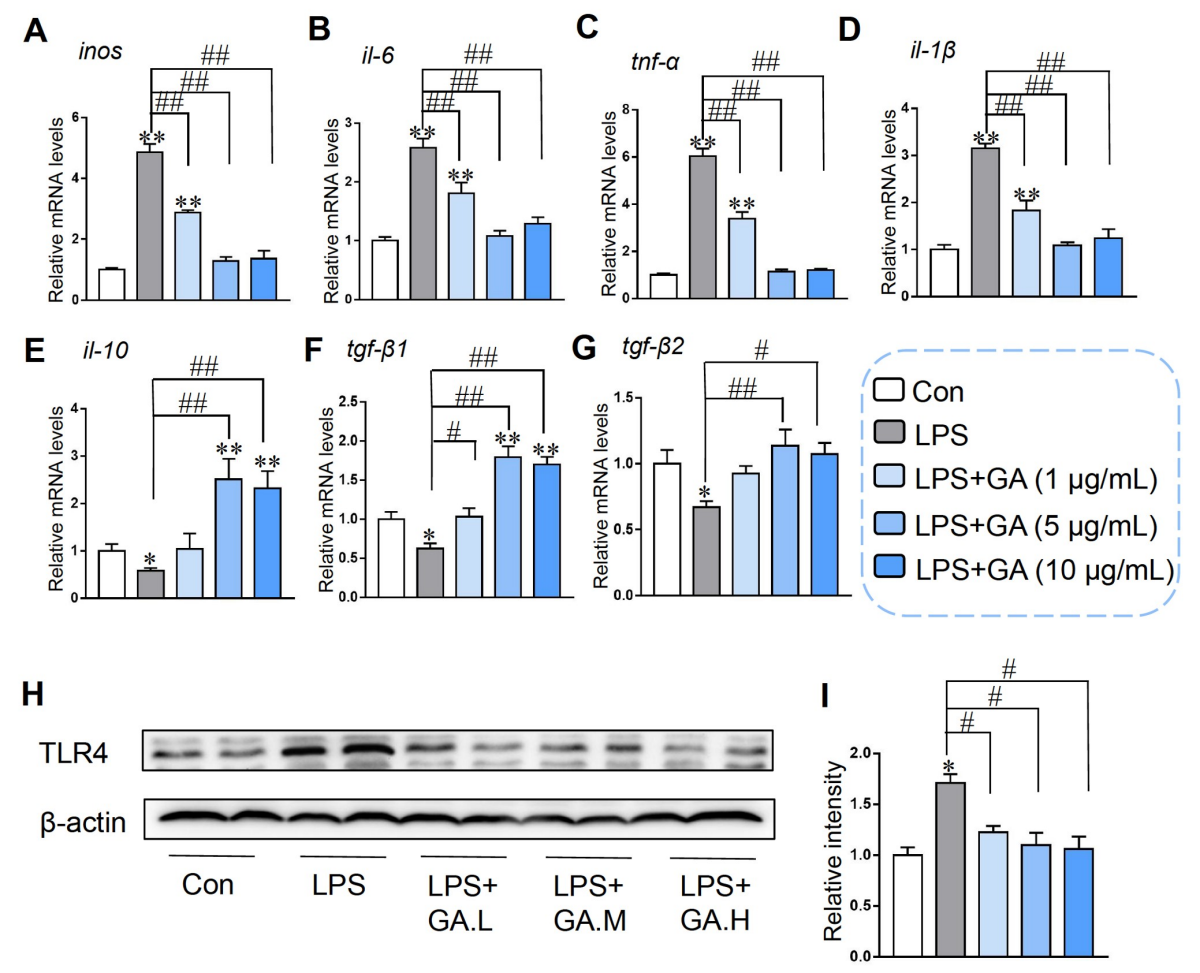
Figure 2 GA inhibited LPS-induced inflammation in Caco-2 cells Caco-2 cells pretreated with or without 1, 5 or 10 μg/mL GA for 24 h were exposed to 10 μg/mL LPS for an additional 4 h. qRT-PCR was utilized to measure the mRNA levels of inflammatory cytokines, such as (A) inos, (B) il-6, (C) tnf-α, (D) il-1β, (E) il-10, (F) tgf-β1, and (G) tgf-β2. The data were standardized to the mRNA expression of β-actin. (H) TLR4 protein expression was assessed via western blot analysis. (I) Relative level of TLR4 protein. The values were standardized to the β-actin level. Data are shown as the mean±SEM, n=6. *P<0.05 and **P<0.01 compared to the control. #P<0.05 and ##P<0.01 compared to the LPS group. GA: gallic acid.

Compared to those in the control group, the *inos*, *il-6*, *tnf-α* and *il-1β* mRNA levels in the LPS treatment group were significantly greater ( Figure 2A‒D). Notably, GA at concentrations of 1, 5, and 10 μg/mL effectively mitigated the LPS-induced increase in the expressions of these proinflammatory genes. As shown in Figure 2E‒G, LPS decreased the mRNA levels of anti-inflammatory cytokines including *il-10*, *tgf-β1* and *tgf-β2*. GA (5 and 10 μg/mL) rescued the inhibition of these cytokines induced by LPS. The protein level of TLR4 was upregulated by LPS, and different concentrations of GA (1, 5 and 10 μg/mL) markedly inhibited the increase in TLR4 expression ( Figure 2H,I). All the data revealed that 5 and 10 μg/mL GA strongly inhibited the LPS-induced inflammatory response in Caco-2 cells.

### GA alleviated intestinal barrier function against LPS-induced damage

**Figure 3:**
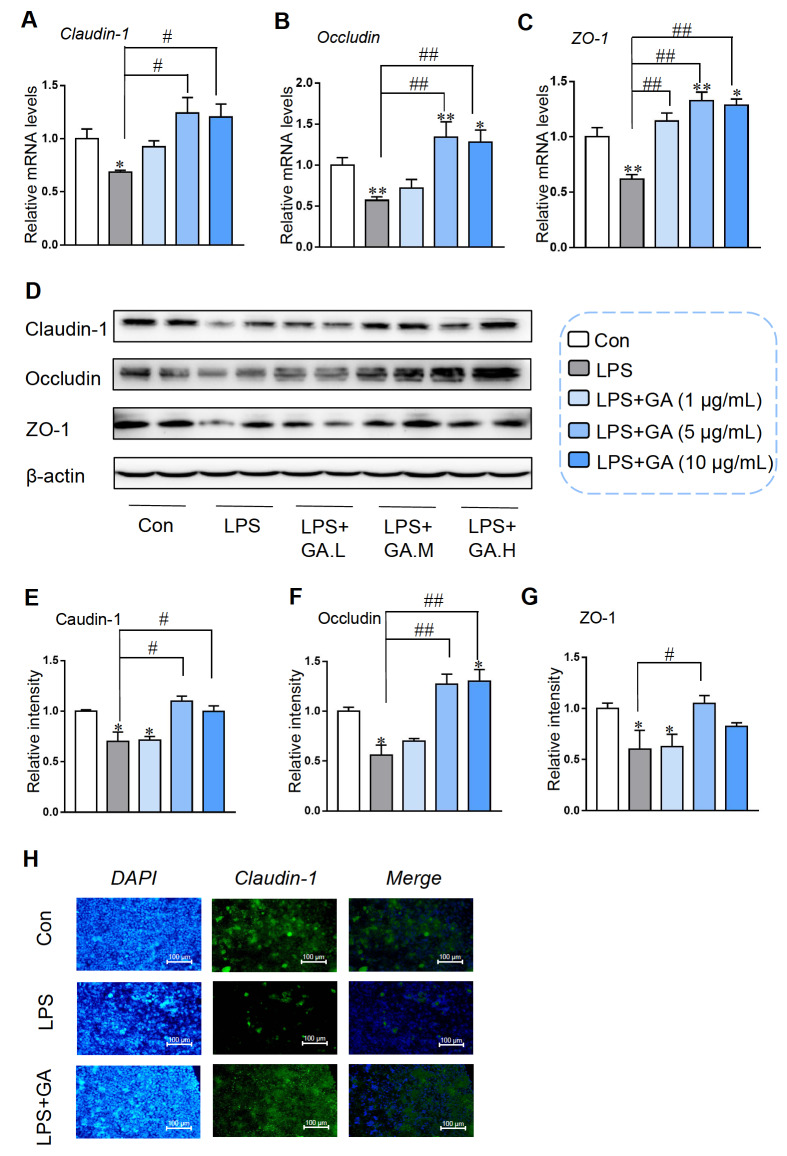
Figure 3 GA protected intestinal barrier function against LPS-induced damage Caco-2 cells were first treated with or without 1, 5, or 10 μg/mL GA for 24 h, followed by 10 μg/mL LPS for 4 h. RT-qPCR was used to measure the mRNA expressions of (A) Claudin-1, (B) Occludin, and (C) ZO-1, and the data were normalized to β-actin expression. (D) Western blot analysis was used to detect the expressions of TJ proteins. Relative levels of (E) Claudin-1, (F) Occludin, and (G) ZO-1. (H) Immunofluorescence staining for Claudin-1 (green) and cell nuclei (blue). Data are shown as the mean±SEM, n=6. *P<0.05 and **P<0.01 compared to the control. #P<0.05 and ##P<0.01 compared to the LPS group. GA: gallic acid.

As crucial members of the TJ protein family, Claudin-1, Occludin, and ZO-1 play indispensable roles in maintaining intestinal barrier function. Similarly, the mRNA levels of *Claudin-1*, *Occludin* and *ZO-1* in Caco-2 cells were decreased in response to LPS treatment, while 5 and 10 μg/mL GA increased the mRNA levels of these proteins in LPS-induced Caco-2 cells ( Figure 3A‒C). Treatment with 5 or 10 μg/mL GA markedly enhanced the protein expressions of Claudin-1 and Occludin ( Figure 3E,F). However, a significant increase in the protein expression of ZO-1 was observed only with 5 μg/mL GA ( Figure 3H). Consistent with the gene expression data, GA restored the LPS-induced reduction in the expressions of these TJ proteins ( Figure 3D‒G). Immunofluorescence staining revealed that the expression level of Claudin-1 in the LPS+GA group was prominently higher than that in the LPS group ( Figure 3H). These results implicated that GA could alleviate LPS-induced intestinal barrier dysfunction.

### GA alleviated LPS-induced Caco-2 cell apoptosis

**Figure 4:**
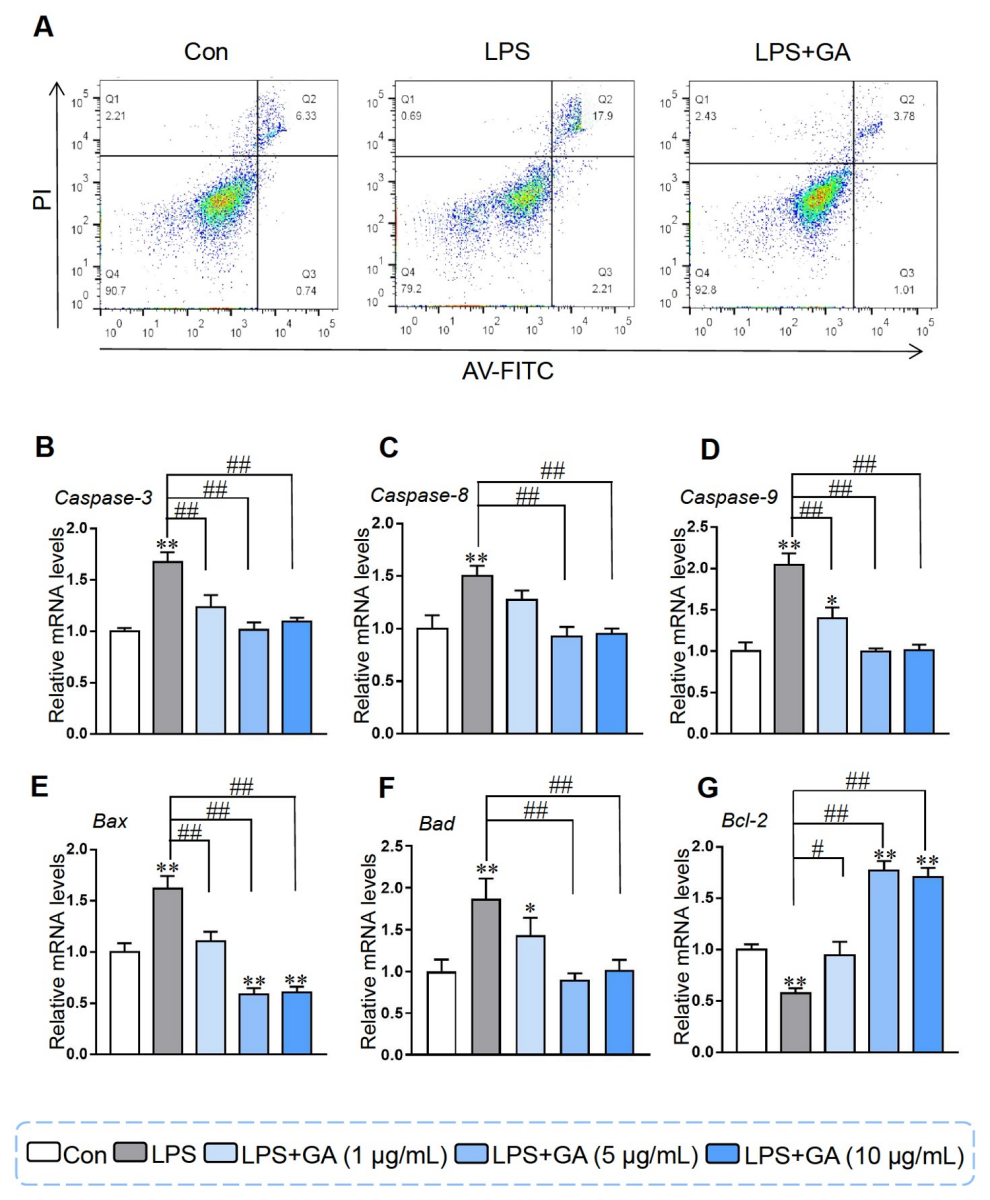
Figure 4 GA alleviated LPS-induced Caco-2 cell apoptosis (A) Caco-2 cells were first treated with 5 μg/mL GA and then with 10 μg/mL LPS in 6-well plates, subsequently stained with Annex V-FITC and analyzed by flow cytometry. qRT-PCR was used to quantify the mRNA expression levels of (B) Caspase-3, (C) Casepase-8, (D) Caspase-9, (E) Bax, (F) Bad, and (G) Bcl-2. Data are presented as the mean±SEM, n=6. *P<0.05 and **P<0.01 compared to the control. #P<0.05 and ##P<0.01 compared to the LPS group. GA: gallic acid.

Compared to those in the control group, the percentages of early apoptotic cells and late apoptotic cells were elevated by nearly 3-fold by LPS treatment. However, treatment with 5 μg/mL GA effectively reduced the percentage of apoptotic cells and restored the percentage to levels comparable to those in the control group ( Figure 4A). According to the flow cytometry results, LPS induced Caco-2 cell apoptosis, and 5 μg/mL GA alleviated this apoptosis ( Figure 4A). The mRNA expression levels of *Caspase-3*, *Caspase-8*, *Caspase-9*, *Bad* and *Bax* increased in response to LPS stimulation ( Figure 4B‒F), while GA counteracted the LPS-induced upregulation of these genes. The expressions of genes corresponding to anti-apoptotic factors, including *Bcl-2*, exhibited the opposite trend ( Figure 4G).

### Effects of GA on ROS levels and antioxidant parameters in Caco-2 cells

**Figure 5:**
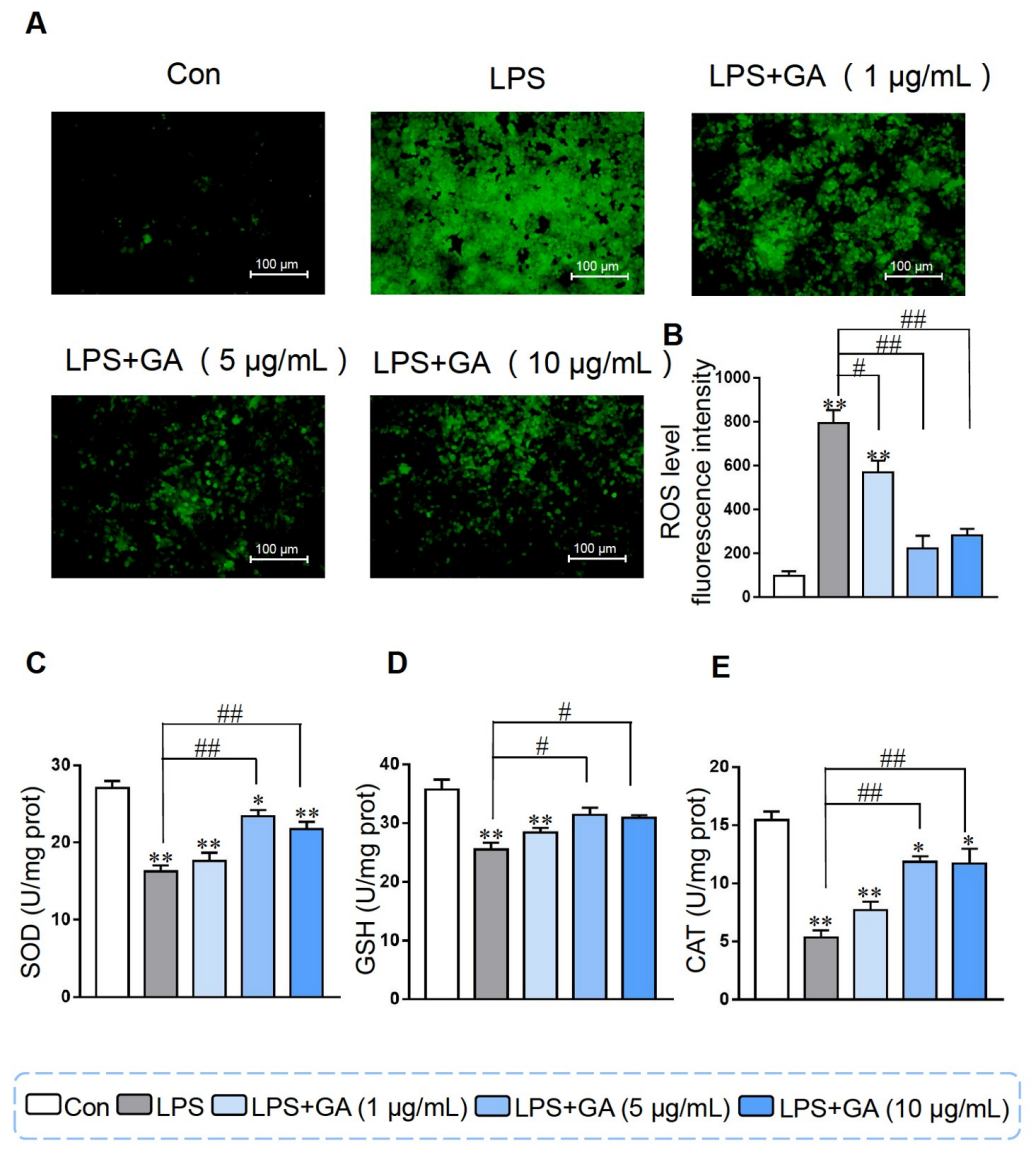
Figure 5 GA alleviated LPS-induced cellular oxidative damage (A) Fluorescence images showing the level of ROS after treatment with different concentrations of GA and subsequent treatment with 10 μg/mL LPS in Caco-2 cells. (B) Quantification of fluorescence. The activity of (C) SOD, (D) GSH, and (E) CAT in Caco-2 cells. Data are shown as the mean±SEM, n=6. *P<0.05 and **P<0.01 compared to the control. #P<0.05 and ##P<0.01 compared to the LPS group. GA: gallic acid.

In contrast to that in the control group, the fluorescence intensity of ROS in the LPS group dramatically increased, and this increase was alleviated by GA treatment in Caco-2 cells. Supplementation with 5 or 10 μg/mL GA dramatically reduced the fluorescence intensity of the ROS ( Figure 5A). As shown in Figure 5B, LPS significantly reduced SOD activity. The addition of 5 or 10 μg/mL GA led to a significant increase in SOD activity, as well as in CAT and GSH levels ( Figure 5C‒E). These results suggested that GA could alleviate oxidative stress induced by LPS in Caco-2 cells.

### GA regulated intestinal inflammation by inhibiting LPS-induced NF-κB activation

**Figure 6:**
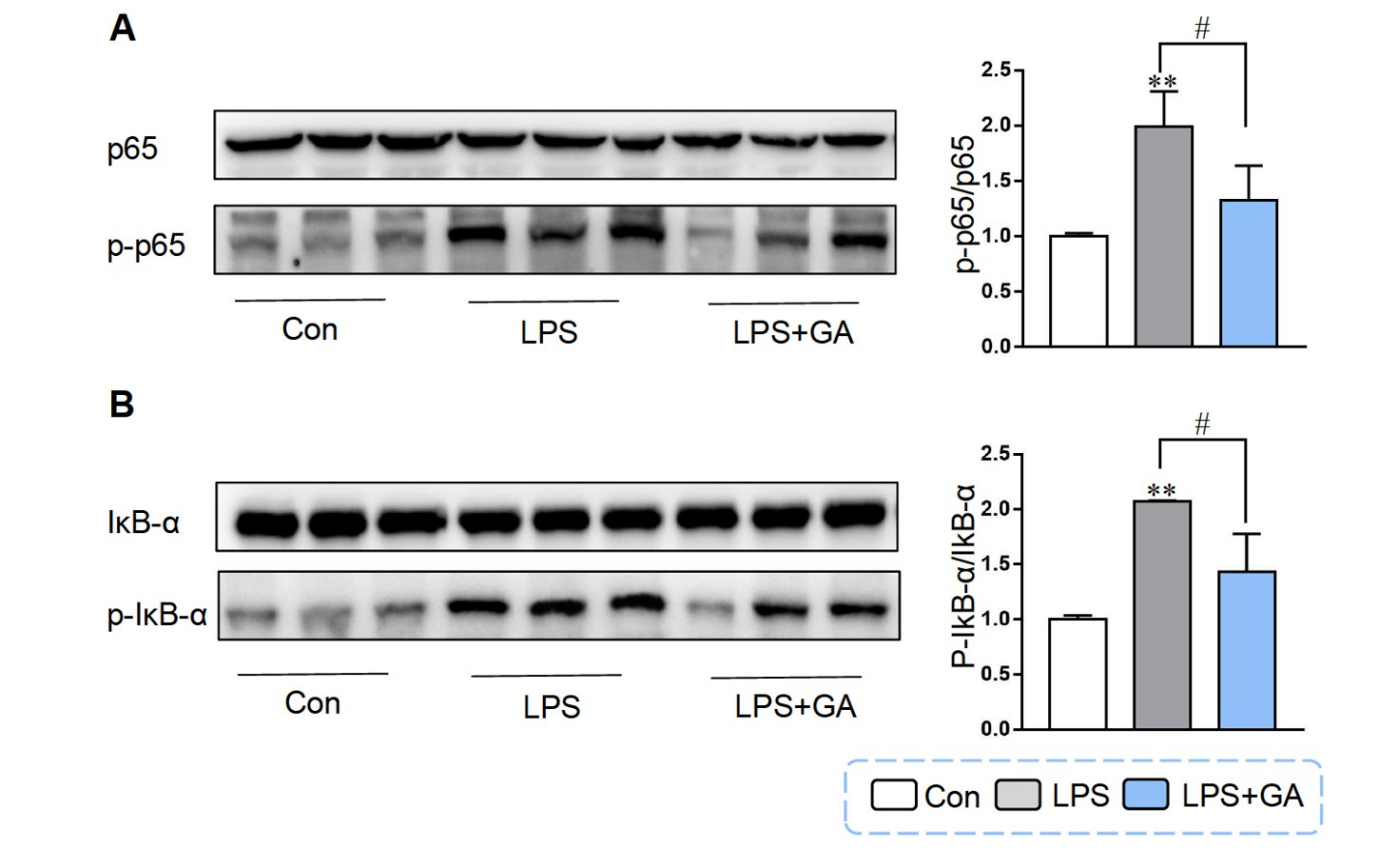
Figure 6 GA regulated intestinal inflammation by inhibiting LPS-induced NF-κB activation Caco-2 cells were treated with 5 μg/mL GA for 24 h and then 10 μg/mL LPS for 4 h for follow-up experiments. (A) Western blot analysis was used to measure the protein expressions of p65 and p-p65. Quantitative analysis of the p-p65/p65 ratio. (B) The protein expression levels of IκB-α and p-IκB-α. Quantitative analysis of the p-IκB-α/IκB-α ratio. Data are shown as the mean±SEM, n=3. **P<0.01 compared to the control. #P<0.05 compared to the LPS group. GA: gallic acid.

To elucidate the molecular mechanism through which GA regulates inflammation, we investigated its impact on the NF-κB signaling pathway. LPS treatment obviously enhanced the phosphorylation of p65 and IκB-α; however, the phosphorylation of p65 and IκB-α was significantly decreased by pretreatment with 5 μg/mL GA for 24 h in Caco-2 cells ( Figure 6). Owing to the suppressive influence of GA on the phosphorylation of p65 and IκB-α, GA may indeed reduce cellular inflammation by inhibiting the NF-κB pathway.

### Effect of GA on the MAPK signaling pathway

**Figure 7:**
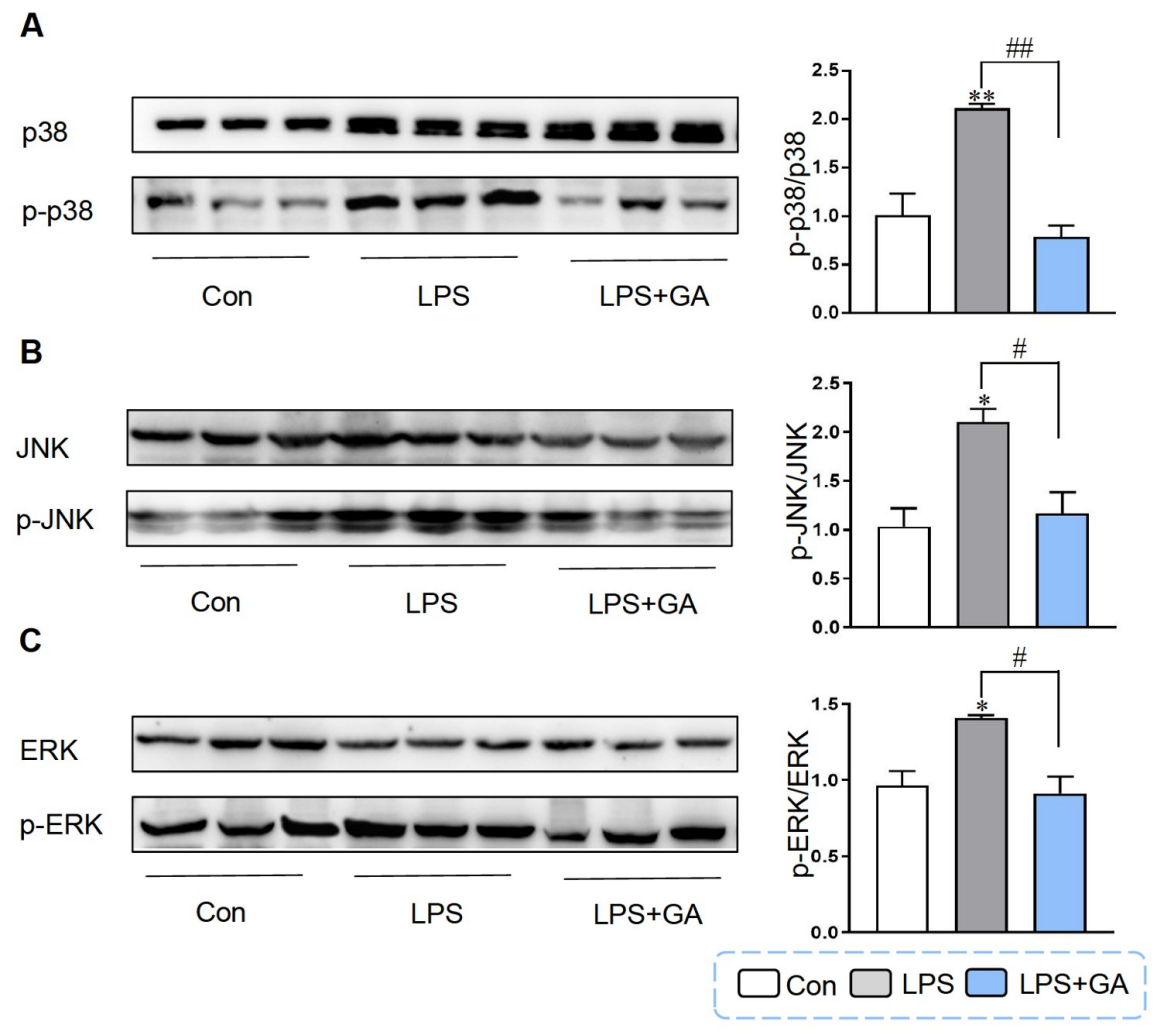
Figure 7 Effect of GA on the MAPK signaling pathway Caco-2 cells were treated with 5 μg/mL GA for 24 h and then incubated with 10 μg/mL LPS for another 4 h for subsequent experiments. (A) Western blot analysis was used to measure the protein expressions of p38 and p-p38. Quantification of the p-p38/p38 ratio. (B) The protein expression levels of JNK and p-JNK. Quantification of the p-JNK/JNK ratio. (C) The protein expression levels of ERK and p-ERK. Quantification of the p-ERK/ERK ratio. Data are shown as the mean±SEM, n=3. *P<0.05 and **P<0.01 compared to the control. #P<0.05 and ##P<0.01 compared to the LPS group. GA: gallic acid.

MAPK is involved in another signaling pathway that participates in the modification of proinflammatory cytokine expression during inflammation [30]. Therefore, we further assessed the p38, JNK and ERK protein levels by western blot analysis. Our results showed that LPS stimulation markedly enhanced the phosphorylation of p38, JNK and ERK in Caco-2 cells, while 5 μg/mL GA weakened the LPS-induced phosphorylation of p38, JNK and ERK ( Figure 7), indicating that MAPK is involved in the regulatory effect of GA on the inhibition of intestinal inflammation induced by LPS.

## Discussion

GA, characterized by its phenolic and carboxylic acid properties, can potentially mitigate inflammation-related diseases by modulating redox status and regulating the intestinal microbiota [ 26, 31]. *In vivo* studies have affirmed the anti-inflammatory effects of GA in conditions such as obesity, diabetes, and colitis [ 27, 29, 32]. Studies have shown that its main anti-inflammatory mechanisms include suppressing the activation of factors involved in the transcription and transduction of signals, reducing the expressions of inflammatory mediators and inhibiting the phosphorylation or transfer of p65-NF-κB [ 33‒ 35]. For example, Li *et al*. [35], reported that GA significantly reduced ROS in RAW264.7 macrophages induced by LPS. These findings highlighted the potent anti-inflammatory properties of GA, as evidenced by its ability to effectively inhibit the activation of the NF-κB pathway in LPS-activated macrophages, leading to a reduction in inflammatory factors such as iNOS, IL-6, and TNF-α [35]. Sripanidkulchai *et al*. [36] revealed that GA, as the main component of the extractive of *Phyllanthus emblica* Linn., could restrain the expressions of COX-2, iNOS, and IL-6 in RAW 264.7 cells [36]. These explorations collectively underscore the potential of GA in alleviating inflammation, suggesting its applicability in the treatment of intestinal diseases. In this study, 5 μg/mL GA was shown to reduce LPS-induced inflammation through regulating TJ protein expressions, inhibiting apoptosis, reducing the generation of ROS, and suppressing the activation of the NF-κB/MAPK pathway in Caco-2 cells. These results further contribute to the growing body of evidence supporting the therapeutic potential of GA in treating intestinal inflammation.

LPS, a component of the outer wall of gram-negative bacteria cells, functions by binding to TLR4, ultimately promoting inflammation [ 37, 38]. LPS can also disrupt the intestinal barrier by regulating inflammatory responses and reducing the expression of TJ proteins [ 38, 39]. TJ proteins, including Claudin, Occludin and ZO-1, play pivotal roles in maintaining intestinal barrier integrity [40]. TJ proteins are critical for conserving the intestinal barrier architecture and regulating the paracellular diffusion of ions and solutes [41]. Studies have linked the decreased expressions of Claudin-1, Occludin, and ZO-1 with gastrointestinal disorders where the intestinal barrier is compromised [ 42, 43]. The damaged intestinal barrier allows some bacteria and antigens to cross the intestinal barrier and stimulate inflammation [44]. Studies have shown that colonic biopsy of patients with IBD results in decreased expressions of TJ proteins, indicating that the regulation of TJ proteins may improve intestinal barrier integrity to reduce the entry of bacteria and other substances into the gut that promote intestinal inflammation [45]. In our study, GA administration counteracted the downregulation of Claudin-1, Occludin and ZO-1 induced by LPS. These immunofluorescence results further confirmed that GA alleviated the LPS-induced reduction in Claudin-1 expression. These findings suggest that GA can potentially alleviate LPS-induced intestinal barrier dysfunction, thereby protecting the integrity of the intestinal barrier.

These data suggest that apoptosis is a key characteristic of the cellular response to LPS. Intestinal barrier damage induced by LPS is associated with increased apoptosis [ 46, 47]. To explore the potential mechanism through which GA repairs intestinal injury and alleviates inflammation, we further investigated the regulatory effect of GA on cell apoptosis. In the present study, the expressions of Bax, Bad, Caspase-3, Caspase-8, and Caspase-9 increased in response to LPS, and GA inhibited the upregulation of these genes induced by LPS. Moreover, GA rescued the inhibitory effect of LPS on the expressions of anti-apoptotic factors such as Bcl-2. Flow cytometric analysis revealed that the number of early and late apoptotic cells increased following LPS exposure, while GA treatment dramatically decreased the proportion of apoptotic cells. Taken together, these results indicate that GA inhibits Caco-2 cell apoptosis induced by LPS.

LPS-induced ROS production is associated with the initiation of apoptosis, and drugs that prevent LPS-induced cell apoptosis also inhibit ROS formation [46]. ROS are recognized factors involved in the pathogenesis of ulcerative colitis (UC), and antioxidants are commonly employed in UC treatment [ 14, 48]. Naturally occurring GA has strong antioxidant activity and can inhibit apoptosis and play a defensive role in organisms [ 49, 50]. In the present study, GA dramatically reduced the production of ROS induced by LPS. ROS accumulation can trigger NF-κB, JNK, P38 and other signaling pathways to enhance inflammation and lead to increased expressions of a series of proinflammatory mediators. Taken together, these results suggest that GA has a strong antioxidant effect and may play a protective role against LPS-induced inflammation by regulating oxidative stress.

TLR4 is an LPS reporter that is able to immediately induce inflammation through activating diverse downstream signaling pathways [51]. Patients with active ulcerative colitis often exhibit high levels of TLR4 expression in the intestinal epithelium, indicating the potential involvement of TLR4 in the progression of UC [52]. The ultimate transcription factor in the TLR4 signaling pathway is NF-κB [52]. Numerous previous explorations have confirmed that the NF-κB signaling pathway persistently plays a considerable role in the development of colitis by regulating the transcription and translation of inflammatory mediators [53]. LPS triggers IκB-α phosphorylation or ubiquitination, leading to IкB-α degradation and enabling NF-κB activation and migration from the cytoplasm to the nucleus. Subsequently, target genes of NF-κB, including *IL-1β*, *TNF-α*, and *iNOS*, are activated [ 53‒ 55]. Pandurangan *et al*. [56] revealed that GA potentially plays a clinical anti-inflammatory role by restraining the activation of p65-NF-κB and IL-6/p-STAT3 ^Y705^. Zhu *et al*. [57] reported that GA suppressed the expressions of the proinflammatory cytokines IL-1/6, TGF-β and TNF-α and promoted the secretion of the anti-inflammatory cytokine IL-4/10 through suppression of the IκB/NF-κB pathway. Most of the above studies demonstrated the impact of GA on colitis through NF-κB in mice. In our study, we investigated the effects of GA on Caco-2 cells and found that the phosphorylation of the p65 and IκB-α proteins induced by LPS was significantly downregulated after pretreatment with 5 μg/mL GA for 24 h. These results indicate that GA rescues LPS-induced inflammation through suppressing the activation of the NF-κB signaling pathway.

MAPKs are a family of serine/threonine protein kinases, including P38, JNK and ERK, that regulate gene expression, cell proliferation, differentiation and other functions, and are implicated in the inflammatory response to LPS stimulation [ 58, 59]. MAPKs also participate in regulating the transcriptional activity of the NF-κB signaling pathway. For example, P38 and JNK can induce the degradation of IκB-α [ 58, 60]. LPS-stimulated cells can activate ERK, JNK and p38 and subsequently act on their substrates to affect the activity of various transcription factors, thus regulating the expressions of diverse cytokines, including TNF, IL-6 and IL-8. Therefore, it can trigger an inflammatory response in the colon through ERK, JNK and p38 [ 61, 62]. In previous studies, several herbal ingredients, such as Ganoderma lucidum polysaccharide, were shown to relieve colitis through the MAPK signaling pathway [63]. Consequently, we wondered whether GA also has potential therapeutic effects on these patients. In the present study, pretreatment with 5 μg/mL GA markedly inhibited the activation of JNK, ERK and p38 caused by LPS. These results indicated that GA prevents the activation of the MAPK signaling pathway stimulated by LPS in Caco-2 cells, thereby exerting anti-inflammatory effects.

In summary, this study revealed that GA significantly reduces the reduction in TJ proteins, inflammatory response, ROS production and apoptosis induced by LPS in Caco-2 cells by inhibiting the activation of the NF-κB and MAPK pathways. Our findings reveal that GA is a promising agent for the treatment of patients with IBD.

## Supporting information

23506Supplementary_Table_S1

## Supplementary Data

Supplementary data is available at *Acta Biochimica et Biophysica Sinica* online.
