# Supplementary material for: Gallic acid attenuates LPS-induced inflammation in Caco-2 cells by suppressing the activation of the NF-κB/MAPK signaling pathway: Gallic acid alleviates the inflammation in Caco-2 cells
Source: Acta Biochim Biophys Sin (Shanghai). 2024 Mar 22;56(6):905–15. doi: 10.3724/abbs.2024008 (PMC11214974; doi:10.3724/abbs.2024008)
Supplement: 23506Supplementary_Table_S1 [file 23506Supplementary_Table_S1.pdf]

Supplementary Table S1. Sequences of primers used in qRT-PCR

Gene	Sequence (5′→3′)
iNOS	Forward CGGCCATCACCGTGTTCCCC<br>Reverse TGCAGTCGAGTGGTGGTCCA
IL-6	Forward CCTTCCAAAGATGGCTGAAA<br>Reverse CAGGGGTGGTTATTGCATCT
TNF-α	Forward CTCTTCTGCCTGCTGCACTTTG<br>Reverse ATGGGCTACAGGCTTGTCACTC
IL-1β	Forward AAACAGATGAAGTGCTCCTTCCAGG<br>Reverse TGGAGAACACCACTTGTTGCTCCA
IL-10	Forward CTGAGGGAGCTCCGTTCTGC<br>Reverse TCGATTGGGGTTGTGGAGTGC
TGF-β1	Forward CTACGAGACCAAGTGCAATCC<br>Reverse AATCGCCAGCCAATTCTCTTT
TGF-β2	Forward GGGCCTGGAGATCAGCGTTC<br>Reverse CGAGAGCTCGCTTTCGTCGT
Claudin-1	Forward AACGCGGGGCTGCAGCTGTTG<br>Reverse GGATAGGGCCTTGGTGTTGGGT
Occludin	Forward TCAGGGAATATCCACCTATCACTTCAG<br>Reverse CATCAGCAGCAGCCATGTACTCTTCAC
ZO-1	Forward CGGTCCTCTGAGCCTGTAAG<br>Reverse GGATCTACATGCGACGACAA
Caspase-3	Forward TGGCGAAATTCAAAGGATG<br>Reverse TAACCCGGGTAAGAATGTGC
Caspase-8	Forward ACATGGACTGCTTCATCTGC<br>Reverse AAGGGCACTTCAAACCAGTG
Caspase-9	Forward GCAGTAACCCCGAGCCAGATG<br>Reverse CCGGAGGAAATTAAAGCAACCAG
Bax	Forward AGGGTTTCATCCAGGATCGAGCAG<br>Reverse ATCTTCTTCCAGATGGTGAGCGAG
Bcl-2	Forward GGTGGGGTCATGTGTGTGG<br>Reverse CGGTTCAGGTACTCAGTCATCC
Bad	Forward TGGACTCCTTTAAGAAGGGAC<br>Reverse CAAGTTCCGATCCCACCAG

GAPDH	Forward CGAGATCCCTCCAAAATCAA<br>Reverse GTCTTCTGGGTGGCAGTGAT
